# Immunisation coverage and factors associated with incomplete immunisation in children under two during the COVID-19 pandemic in Sierra Leone

**DOI:** 10.1186/s12889-023-17534-2

**Published:** 2024-01-10

**Authors:** Myrte Wassenaar, Augustin E. Fombah, Haily Chen, Kwabena Owusu-Kyei, Julian Williams, Joe-Henry C. Sunders, Mireia Llach, Llorenç Quinto, Tom Sesay, Mohamed Samai, Clara Menéndez, Raquel González

**Affiliations:** 1 ISGlobal, Hospital Clínic - Universitat de Barcelona, Barcelona, Spain; 2 University Medical Center Utrecht - Utrecht University, Utrecht, the Netherlands; 3 Ministry of Health, Freetown, Sierra Leone; 4 Manhiça Health Research Center, Manhiça, Mozambique; 5 College of Medicine and Allied Health Sciences, University of Sierra Leone, Freetown, Sierra Leone; 6 Directorate of Reproductive Child Health, Ministry of Health, Freetown, Sierra Leone; 7 Directorate of Research and Training, Ministry of Health, Freetown, Sierra Leone

**Keywords:** Childhood immunisation, Routine immunisation coverage, Household survey, Sierra Leone

## Abstract

**Background:**

Routine childhood immunisation is one of the most important life-saving public health interventions. However, many children still have inadequate access to these vaccines and millions remain (partially) unvaccinated globally. As the COVID-19 pandemic disrupted health systems worldwide, its effects on immunisation have become apparent. This study aimed to estimate routine immunisation coverage among children under two in Sierra Leone and to identify factors associated with incomplete immunisation during the COVID-19 pandemic.

**Methods:**

A cross-sectional household survey was conducted in three districts in Sierra Leone: Bombali, Tonkolili and Port Loko. A three-stage cluster sampling method was followed to enrol children aged 10–23 months. Information regarding immunisation status was based on vaccination cards or caretaker’s recall. Using WHO’s definition, a fully immunised child received one BCG dose, three oral polio vaccine doses, three pentavalent vaccine doses and one measles-containing vaccine dose. Following the national schedule, full immunisation status can be achieved at 9 months of age. Data were weighted to reflect the survey’s sampling design. Associations between incomplete immunisation and sociodemographic characteristics were assessed through multivariable logistic regression.

**Results:**

A total of 720 children were enrolled between November and December 2021. Full vaccination coverage was estimated at 65.8% (95% CI 60.3%-71.0%). Coverage estimates were highest for vaccines administered at birth and decreased with doses administered subsequently. Adjusting for age, the lowest estimated coverage was 40.7% (95% CI 34.5%-47.2%) for the second dose of the measles-containing vaccine. Factors found to be associated with incomplete immunisation status were: living in Port Loko district (aOR = 3.47, 95% CI = 2.00-6.06; *p*-value < 0.001), the interviewed caretaker being Muslim (aOR = 1.94, 95% CI = 1.25–3.02; *p*-value = 0.015) and the interviewed caretaker being male (aOR = 1.93, 95% CI = 1.03–3.59, *p*-value = 0.039).

**Conclusion:**

Though full immunisation coverage at district level improved compared with pre-pandemic district estimates from 2019, around one in three surveyed children had missed at least one basic routine vaccination and over half of eligible children had not received the recommended two doses of a measles-containing vaccine. These findings highlight the need to strengthen health systems to improve vaccination uptake in Sierra Leone, and to further explore barriers that may jeopardise equitable access to these life-saving interventions.

**Supplementary Information:**

The online version contains supplementary material available at 10.1186/s12889-023-17534-2.

## Introduction

Immunisation is one of the most important and successful public health interventions to date [1]. It remains crucial in protecting children from the morbidity and mortality caused by vaccine-preventable diseases (VPDs). Through routine vaccination, an estimated 5.1 million lives per year will be saved between 2021 and 2030 [2]. Yet, especially in low-income settings many children have insufficient access to vaccines, leaving almost one in five children (partially) unvaccinated and exposed to preventable diseases, disability and premature death [1, 3].

To ensure equal access to recommended vaccines for all children worldwide, the World Health Organization (WHO) established the Expanded Programme on Immunisation (EPI) in 1974 [4]. Initially, the EPI targeted six major infectious diseases: diphtheria, pertussis, poliomyelitis, tetanus, tuberculosis and measles [4]. In Sierra Leone, since EPI implementation in 1978, the targeted VPDs have been expanded by adding vaccines against yellow fever, hepatitis B, Haemophilus influenzae type b, rotavirus and pneumococcal disease [5, 6]. Improvements in immunisation coverage have been made following extensive efforts to rebuild the country’s health system after a decade-long civil war and after the 2014–2015 Ebola virus disease outbreak [7]. According to Sierra Leone’s Demographic and Health Surveys (DHS), the proportion of children aged 12–23 months that had received all vaccinations appropriate for their age rose from 31% in 2008 to 58% in 2013, and subsequently decreased to 49% in 2019 [8, 9]. In the latter year, 2% had not received any vaccinations. Suboptimal immunisation coverage therefore remains a major public health challenge in the country. [9] After the 2019 DHS was conducted, the COVID-19 pandemic led to another disruption of essential health services. The impact of the pandemic on EPI coverage is unclear since vaccination coverage surveys have not been conducted during this period.

As the COVID-19 pandemic put a strain on health systems around the world, its effect on global infant immunisation coverage became apparent [10, 11]. In 2021, approximately 25 million children missed out on essential vaccines, an estimated six million more than in 2019 [11]. Of these, 18.2 million had not received any vaccinations at all [3]. Consequently, the world has been confronted with a surge in outbreaks of VPDs, including polio, yellow fever and measles [12].

Given the urgency to improve immunisation coverage, WHO launched a global strategy, the Immunisation Agenda 2030, that strives towards equal access to life-saving vaccines for everyone, everywhere, in the coming decade [1]. As part of this strategy, WHO promotes vaccination coverage monitoring at subnational levels. Achieving equity in vaccination uptake requires an understanding of cultural, geographic, socioeconomic and systemic barriers to immunisation. Reporting and analysing vaccination coverage at a local or regional level facilitates the identification of disparities in uptake. Such findings provide critical information needed to prioritise and tailor strategies to close immunisation gaps.

The objective of this study was to determine routine immunisation coverage among children aged 10–23 months living in selected areas of Sierra Leone during the COVID-19 pandemic, and to identify risk factors associated with incomplete immunisation status in this age group.

## Methods

### Study area

The study was conducted in three districts of Sierra Leone’s Northern and North West Provinces: Bombali, Tonkolili and Port Loko. The three districts were chosen based on malaria prevalence data according to the 2016 Malaria Indicator Survey [13]. Although prevalence in Bombali was slightly lower, the district was selected for logistical reasons.

**Fig. 1:**
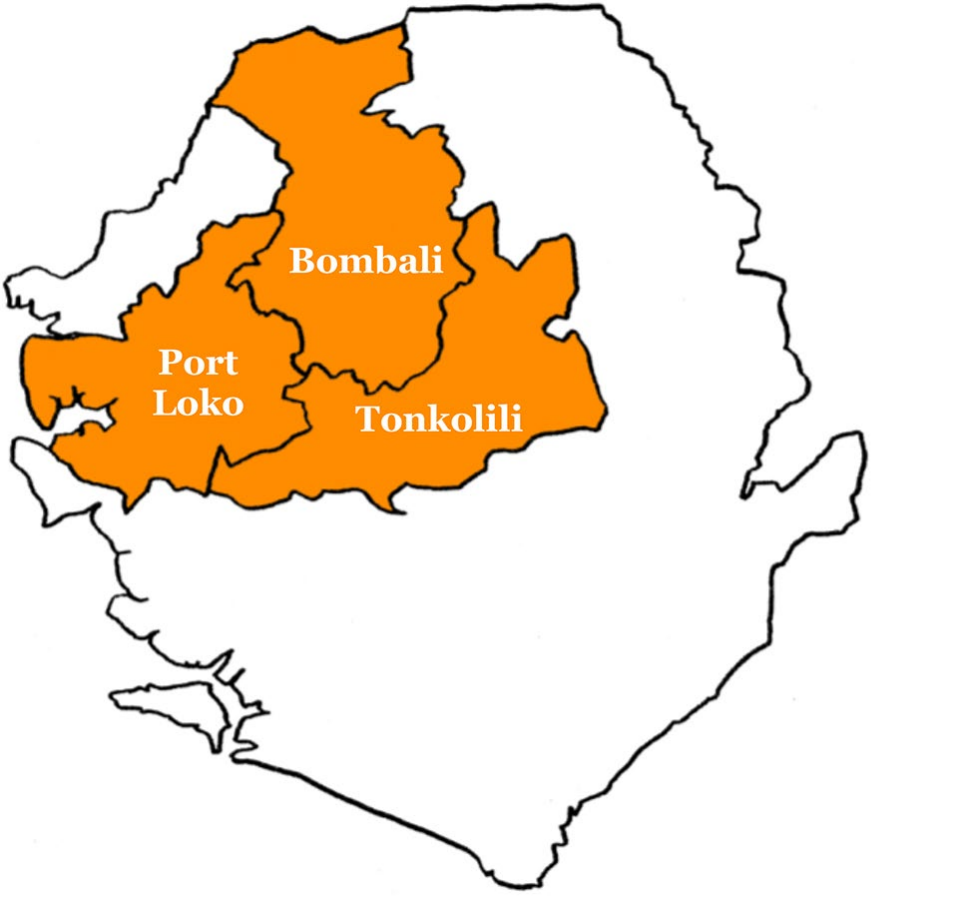
Map of HHS districts in Sierra Leone

Previous estimates of full immunisation coverage among children aged 12–23 months were 63% in Bombali, 49% in Tonkolili and 44% in Port Loko, according to Sierra Leone’s 2019 DHS (Fig. 1) [9]. Notably, Port Loko held the lowest immunisation coverage at district level in the country. According to the 2015 national census, Bombali had a total population of around 607k, of whom 105k were children under the age of 5. For Tonkolili and Port Loko, these population figures are respectively 531k (103k) and 615k (118k) [14].

### Study design and population

As part of the MULTIPLY-project (*MULTIple doses of Intermittent Preventive Treatment during Infancy –IPTi– Proposal: a Lifesaving high Yield intervention*), a cross-sectional community-based household survey (HHS) was designed to estimate baseline malaria prevalence and Perennial Malaria Chemoprevention (PMC; formerly named IPTi) coverage among children living in the selected project areas [15]. This implementation research project is currently ongoing and aims to maximise the delivery and uptake of PMC [16]. Inclusion criteria for the HHS were: (i) age 10–23 months and (ii) residence in a project district at the time of the survey. Caretakers of eligible children were interviewed after they had agreed to participate in the survey by signing an informed consent form.

### Sample size

The primary objective of the HHS was to determine the coverage of three doses of PMC. A minimum sample size of 710 children was deemed to be needed, considering the latest available estimate of 32.2% PMC coverage, precision of 5%, significance level of 5%, a 10% increase to account for non-response and a design effect of 2 to adjust for the effect of cluster sampling [17, 18].

### Sampling procedures

A three-stage cluster sampling method was followed [19]. In the first stage, clusters were randomly selected using probability proportional to size. The population data used for this survey was an estimated projection based on 2015 census data [14]. After cluster mapping, households were visited subsequently following a list of enumerated households, the first being selected using a random number table. If there were two or more eligible children living in a selected household, the table was used to select one child in the third sampling stage.

### Data collection

Data was collected on electronic devices using REDCap software and a pre-tested questionnaire, as recounted in more detail by Fombah et al. [19, 20]. The vaccines that surveyed children received were copied from immunisation cards. If this card was unavailable, vaccine history was based on caretakers’ recall.

### Outcomes, definitions and data analysis

Children were categorised as fully immunised, partially immunised or unimmunised (zero-dose). Similar to Sierra Leone’s DHS, the WHO definition of a fully immunised child was used: an infant who has received one dose of bacille Calmette-Guerin (BCG) vaccine, three doses of oral polio vaccine (OPV; excluding the dose given at birth), three doses of pentavalent vaccine and one dose of measles-containing vaccine (MCV) [9, 21]. In this study, these are referred to as basic vaccinations. Full immunisation status can be achieved at 9 months of age in Sierra Leone [5]. Unimmunised, i.e., zero-dose, children will have received none of the previously listed vaccines, whereas partially immunised children will have missed at least one but not all of these doses. Immunisation coverage was defined as the proportion of children who were fully immunised by the time of the interview. Incomplete immunisation status was defined by grouping zero-dose and partially immunised children. Coverage rates of individual vaccines were defined as the proportion of children to whom this vaccine had been administered at any given time before the survey. Secondly, full immunisation coverage was determined considering all age-appropriate vaccinations from birth until 9 months of age following Sierra Leone’s EPI schedule. In addition to the above-mentioned vaccines, these children received a birth dose of OPV, three doses of pneumococcal vaccine, two doses of rotavirus vaccine, one dose of inactivated poliovirus vaccine (IPV) and one dose of yellow fever vaccine. Vitamin A, PMC, deworming and the second MCV dose (MCV2) were not included in this definition. Individual coverage rates of these doses are reported. As deworming and the second vitamin A supplement are administered at 12 months of age following Sierra Leone’s EPI schedule, coverage of these doses is determined amongst the subgroup of children aged 13 months or older, allowing for one month of delay in uptake. Similarly, because MCV2 is administered at 15 months, its coverage is determined considering enrolled children aged 16 months or older.

Survey weights were applied to account for the survey’s multi-stage cluster sampling design (Appendix 1). Weights for all three sampling stages were calculated using data collected by Statistics Sierra Leone in the 2015 national census [14].

Standard descriptive statistics were used to summarise baseline child, caretaker and household characteristics. Categorical variables were reported in frequencies and weighted percentages. Continuous variables were described using the weighted mean and standard deviation (SD). Vaccination card availability was reported. Full, partial and zero-dose immunisation coverage rates and coverage rates of individual vaccines were determined per survey area and at district level. Regarding type of locality, this study followed the definition of Sierra Leone’s national statistics office, where a locality with ≥ 2000 inhabitants is classified as urban and a locality of < 2000 inhabitants is classified as rural.

Univariate and multivariable logistic regression analyses were performed to assess the association between sociodemographic factors and incomplete immunisation status. Based on previous literature, the following predictive factors were included in the models: district of residence, locality (rural versus urban), child’s age and sex, caretaker’s age, sex, highest level of education, literacy level, main type of income, marital status and religion, and the sex of the household head [22, 23]. Two multivariable logistic regression models were constructed, with incomplete immunisation status based on the WHO definition and on Sierra Leone’s EPI schedule. Crude and adjusted odds ratios (ORs) and 95% confidence intervals (CIs) were reported. *P*-values of less than 0.05 were considered statistically significant.

A subgroup analysis was performed to enable comparisons with other surveys by conforming to the eligibility criteria proposed by the WHO reference manual on vaccination coverage surveys [24]. Coverage rates were determined and logistic regression analyses were conducted amongst children aged 12–23 months.

Statistical analyses were performed using Stata/BE 17.0 [25].

## Results

**Fig. 2:**
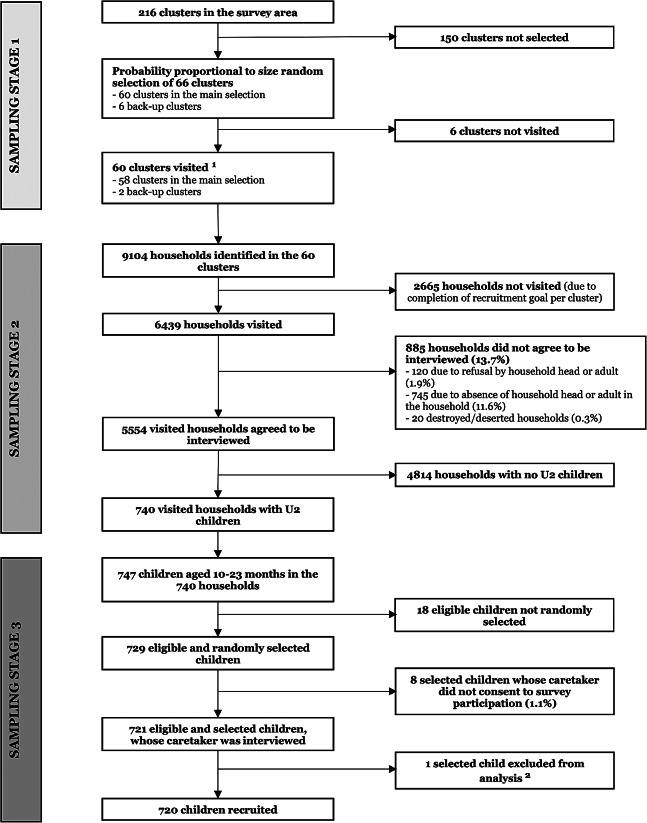
Flowchart of the survey U2 = under two years of age ^1^ To provide the list of clusters within project areas and the maps of the randomly selected clusters, Statistics Sierra Leone used information collected during the national census in 2015. Due to the year difference, two randomly selected clusters were no longer in the project areas and had to be substituted by back up clusters ^2^ One initially recruited child was later excluded from the analysis, due to their recent relocation from Liberia to a survey cluster, which was not an exclusion criterion at the time of data collection. They had not followed the EPI schedule of Sierra Leone but instead had been vaccinated according to the slightly different program in Liberia, and therefore did not meet the study objectives

A total of 720 children aged 10–23 months were recruited from November to December 2021 and included in the analysis: 288 in Bombali, 168 in Tonkolili and 264 in Port Loko (Fig. 2). In 86.3% of the 6,439 visited households, an adult consented to provide information on the children living there. Eight out of 729 selected children’s caretakers did not consent to survey participation. The survey’s non-response rate was, therefore, 13.7% at household level and 1.1% at participant level.

### Sociodemographic characteristics

Baseline characteristics of enrolled children are described in Table 1. The mean (SD) age of children was 17.2 (4.1) months and 50.4% were female. 40% resided in Bombali, 36.7% in Port Loko, and 23.3% in Tonkolili. The location of their households was classified as rural, as opposed to urban, in 75.0% of cases. Of all interviewed caretakers, 87.0% were female and 77.6% were the mother of the enrolled child.

**Table 1:** Baseline characteristics of study children

		n or Mean ± SD	Proportion (%)
**District (N = 720)**	Bombali	288	40.0
	Port Loko	264	36.7
	Tonkolili	168	23.3
**Locality (N = 720)**	Rural	540	75.0
	Urban	180	25.0
**Child’s age in months (N = 720)**		16.68 ± 4.41	-
**Child’s sex (N = 720)**	Female	363	50.4
	Male	357	49.6
**Caretaker’s age in years** ^**1**^ **(N = 718)**		32.4 ± 12.5	-
**Caretaker’s sex** ^**1**^ **(N = 717)**	Female	624	87.0
	Male	93	13.0
**Caretaker’s relationship with the child (N = 720)**	Mother	556	77.6
	Father	70	9.8
	Grandparent	71	9.9
	Aunt or Uncle	19	2.7
	Other	1	1.4
**Caretaker’s highest level of education 1 (N = 718)**	Never attended school	395	55.0
	Primary	78	10.9
	JSS (Middle School)	103	14.4
	SSS (High School)	106	14.8
	Tertiary	36	5.0
**Caretaker’s main type of income 1 (N = 717)**	No salary	79	11.0
	Self-employment	611	85.2
	Paid employment	27	3.8
**Is the caretaker able to read and write? (N = 720)**	Fully literate	96	13.3
	Partially literate	105	14.6
	Illiterate	519	72.1
**Caretaker’s marital status** ^**1**^ **(N = 715)**	Married or in union	578	80.8
	Single (never married)	82	11.5
	Separated or divorced	24	3.4
	Widowed	31	4.3
**Caretaker’s religion** ^**2**^ **(N = 720)**	Muslim	508	70.6
	Christian	210	29.2
	None	2	0.3
**Caretaker’s ethnic group (N = 720)**	Temne	449	62.6
	Limba	120	16.7
	Soso	17	2.4
	Fula	11	1.5
	Mende	11	1.5
	Loko	73	10.1
	Madingo	14	1.9
	Krio	2	0.3
	Sherbro	2	0.3
	Bullom	1	0.1
	Other	20	2.8
**Is the caretaker the household head? (N = 720)**	Yes	365	50.7
	No	355	49.3
**Household head’s sex** ^**1**^ **(N = 719)**	Female	392	54.5
	Male	327	45.5

^1^ Missing values: caretaker’s age = 2; caretaker’s sex = 3; caretaker’s highest level of education = 2; caretaker’s main type of income = 3; caretaker’s marital status = 5, household head’s sex = 1

^2^ Other religion 0/720

### Vaccination coverage

Following the WHO definition of a fully immunised child, 65.8% (95% CI 60.3%-71.0%) of children were fully vaccinated (Table 2). More than half (54.4%; 95% CI 49.1%-59.7%) had received all age-appropriate vaccinations from birth until nine months of age following Sierra Leone’s EPI schedule. Of the 720 enrolled children, 14 (1.9%; 95% CI 1.0%-3.7%) had not received any routine childhood vaccinations.

**Table 2:** Immunisation status of children aged 10–23 months in project districts in Sierra Leone

	Survey districts			
	Bombali n/N (%); [95% CI]	Tonkolili n/N (%); [95% CI]	Port Loko n/N (%); [95% CI]	Survey area n/N (%); [95% CI]
**Fully immunised** (according to WHO definition)^1^	228/288 (79.2); [71.6–85.2]	118/168 (70.2); [59.8–78.9]	128/264 (48.5); [41.2–55.8]	474/720 (65.8); [60.3–71.0]
**Fully immunised** (all age-appropriate vaccines from birth to 9 months following Sierra Leone’s EPI schedule)^2^	193/288 (67.0); [59.4–73.8]	87/168 (51.8); [40.6–62.8]	112/264 (42.4); [35.2–50.0]	392/720 (54.4); [49.1–59.7]
**Partially immunised** (according to WHO definition)^1^	59/288 (20.5); [14.5–28.1]	50/168 (29.8); [21.1–40.2]	123/264 (46.6); [39.5–53.8]	232/720 (32.2); [27.4–37.5]
**Partially immunised** (some but not all age-appropriate vaccines from birth to 9 months following Sierra Leone’s EPI schedule)^2^	94/288 (32.6); [25.8–40.3]	81/168 (48.2); [37.2–59.4]	139/264 (52.7); [45.5–59.7]	314/720 (43.6); [38.5–48.8]
**Unimmunised** (zero-dose)	1/288 (0.4); [0.05–2.4]	0/168 (0.0)	13/264 (4.9); [2.7-9.0]	14/720 (1.9); [1.0-3.7]

^1^ WHO definition of a fully immunised child: a child who received 1 dose of bacille de Calmette-Guérin (BCG) vaccine for tuberculosis, 3 doses of oral polio vaccine (OPV), 3 doses of pentavalent vaccine and 1 dose of measles-containing vaccine (MCV). Partially immunised: defined as a child who received at least one but not all the previously listed vaccines

^2^ Following Sierra Leone’s national EPI schedule, a fully immunised child received all vaccines scheduled from birth to 9 months. In addition to the previously listed vaccines, this child received a birth dose of OPV, three doses of pneumococcal vaccine, two doses of rotavirus vaccine, one dose of inactivated poliovirus vaccine and one dose of yellow fever vaccine. Partially immunised: defined as a child who received at least one but not all recommend vaccines following this schedule

At district level, full vaccination coverage was found to be highest in Bombali: 79.2% (95% CI 71.6%-85.2%) of participants had received all basic vaccinations and 67.0% (95% CI 59.4%-73.8%) had received all age-appropriate vaccinations. In Tonkolili, all basic vaccinations were administered to 70.2% (95% CI 59.8%-78.9%) of children and all age-appropriate vaccinations to 51.8% (95% CI 40.6%-62.8%) of children. Port Loko had the lowest immunisation coverage, with 48.5% (95% CI 41.2%-55.8%) of children having received all basic vaccinations and 42.4% (95% CI 35.2%-50.0%) of children all age-appropriate vaccinations. In this district, close to one in 20 children were unimmunised (4.9%; 95% CI 1.0%-3.7%).

**Fig. 3:**
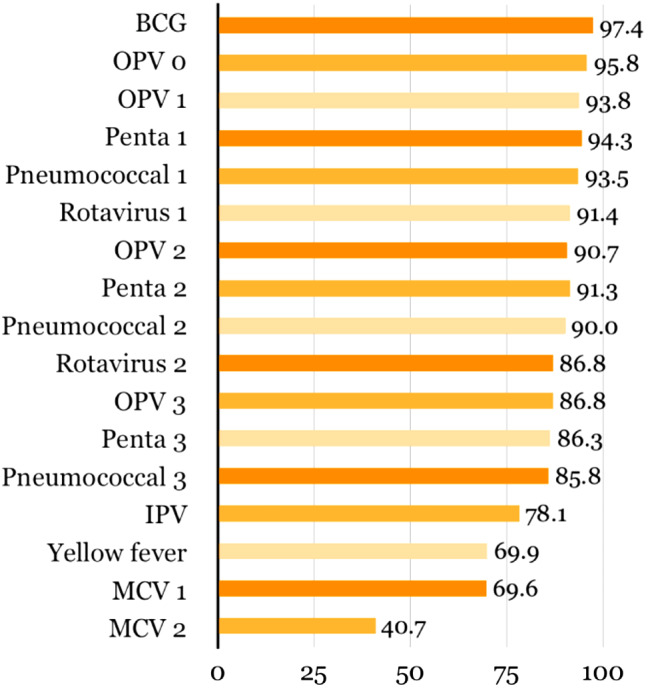
Proportion of children aged 10–23 months vaccinated at any time before the survey^1,2^ BCG = bacille Calmette-Guerin; Penta = pentavalent vaccine; IPV = inactivated polio vaccine; MCV = measles containing vaccine; OPV = oral polio vaccine ^1^ Ordered following Sierra Leone’s EPI schedule ^2^ MCV2: coverage among participants aged 16 months or older, allowing one month of delay

Coverage of individual vaccines was the highest for BCG (97.4%; 95% CI 95.6%-98.4%), administered at birth. For the five vaccines that require multidose administration, the first dose was administered most frequently (Table 3; Fig. 3). The vaccine with the lowest coverage was the second MCV dose administered around 15 months of age. Among children aged 16–23 months, 40.7% (95% CI 34.5%-47.2%) had received the vaccine, whereas the first dose was administered to 69.6% (95% CI 64.1%-74.5%) of children around 9 months.

**Table 3:** Coverage per EPI contact by source of information

Recommended timing of EPI contact	Vaccine	Children aged 10–23 months vaccinated at any time before the HHS, according to:		
		Vaccination card n/N (%)	Caretaker’s report n/N (%)	Total n/N (%); [95% CI]
**At birth**	BCG	564/720 (78.3)	137/720 (19.0)	701/720 (97.4); [95.6–98.4]
	OPV 0	553/720 (74.0)	137/720 (19.0)	690/720 (95.8); [93.4–97.4]
**At 6 weeks**	OPV 1	548/720 (76.8)	127/720 (17.6)	675/720 (93.8); [90.4–96.0]
	DTP-HepB-Hib (Pentavalent) 1	549/720 (76.3)	130/720 (18.1)	679/720 (94.3); [91.1–96.4]
	Pneumococcal 1	545/720 (75.7)	128/720 (17.8)	673/720 (93.5); [90.1–95.7]
	Rotavirus 1	529/720 (73.5)	129/720 (17.9)	658/720 (91.4); [87.7–94.1]
**At 10 weeks**	OPV 2	527/720 (73.2)	126/720 (17.5)	653/720 (90.7); [87.0-93.4]
	DTP-HepB-Hib (Pentavalent) 2	531/720 (73.8)	126/720 (17.5)	657/720 (91.3); [87.6–93.9]
	Pneumococcal 2	522/720 (72.5)	126/720 (17.5)	648/720 (90.0); [86.3–92.8]
	Rotavirus 2	498/720 (69.2)	127/720 (17.6)	625/720 (86.8); [83.0-89.9]
	PMC 1	421/720 (58.5)	153/720 (21.3)	574/720 (79.7); [74.7–83.9]
**At 14 weeks**	OPV 3	503/720 (69.9)	122/720 (16.9)	625/720 (86.8); [82.8–90.0]
	DTP-HepB-Hib (Pentavalent) 3	499/720 (69.3)	122/720 (16.9)	621/720 (86.3); [82.4–89.4]
	Pneumococcal 3	498/720 (69.2)	120/720 (16.7)	618/720 (85.8); [81.9–89.0]
	IPV	432/720 (60.0)	130/720 (18.1)	562/720 (78.1); [73.6–81.9]
	PMC 2	452/720 (62.8)	125/720 (17.4)	577/720 (80.1); [75.7–83.9]
**At 6 months**	Vitamin A	466/720 (64.7)	119/720 (16.5)	585/720 (81.3); [76.4–85.3]
**At 9 months**	Yellow fever	407/720 (56.5)	96/720 (13.3)	503/720 (69.9); [64.4–74.8]
	MCV 1	400/720 (55.6)	101/720 (14.0)	501/720 (69.6); [64.1–74.5]
	PMC 3	328/720 (45.6)	92/720 (12.8)	420/720 (58.3); [53.0-63.5]
**At 12 months**	De-worming^1^	259/571 (45.4)	72/571 (12.6)	331/571 (58.0); [52.1–63.6]
	Vitamin A^1^	260/571 (45.5)	74/571 (13.0)	334/571 (58.5) [52.7–64.0]
**At 15 months**	MCV 2^2^	128/442 (29.0)	52/442 (11.8)	180/442 (40.7) [34.5–47.2]

BCG = bacille Calmette-Guérin; DTP = diphtheria-tetanus-pertussis; HepB = hepatitis B; Hib = Haemophilus influenzae type b; PMC = perennial malaria chemoprevention; IPV = inactivated polio vaccine; MCV = measles-containing vaccine; OPV = oral polio vaccine

^1^ Among participants aged 13 months or older, allowing one month of delay

^2^ Among participants aged 16 months or older, allowing one month of delay

### Vaccination card availability

Reported immunisation coverage rates were based on presented vaccination cards or, if absent, on caretakers’ recall. A vaccination card was presented during 80.0% of interviews (Table 4). Reported reasons for unavailability were: the card was lost or damaged in 33.9% of cases, 4.0% of caretakers was not given a card for their child, and someone other than the interviewed caretaker kept the card in 47.6% of cases. Reasons other than the three previously mentioned accounted for 14.5% of unavailable vaccination cards. Of the latter, the caretaker forgot or purposely left the card elsewhere in 10 out of 18 cases, three of which were due to relocation.

**Table 4:** Vaccination card availability

	Survey districts			
	Bombali n/N (%); [95% CI]	Tonkolili n/N (%); [95% CI]	Port Loko n/N (%); [95% CI]	Survey area n/N (%); [95% CI]
**Vaccination card available and seen during interview**	245/288 (85.1); [79.9–89.1]	147/168 (87.5); [81.6–91.7]	184/264 (69.7); [62.9–75.7]	576/720 (80.0); [76.1–83.4]
**Vaccination card not seen during interview**	43/288(14.9); [10.9–20.1]	21/168 (12.5); [8.3–18.4]	80/264 (30.3); [24.3–37.1]	144/720 (20.00); [16.6–23.9]
**Reasons for unavailability vaccination card** ^1^				
The card is lost/damaged	13/41 (31.7)	6/20 (30.0)	23/63 (36.5)	42/124 (33.9)
The caretaker was not given one	3/41 (7.3)	2/20 (10.0)	0/63 (0.0)	5/124 (4.0)
Someone else keeps it	21/41 (51.2)	10/20 (50.0)	28/63 (44.4)	59/124 (47.6)
Other	4/41 (9.8)	2/20 (10.0)	12/63 (19.1)	18/124 (14.5)

^1^ No data for 20 cases: 9 children never attended EPI services and in 11 cases a vaccination card was available but not seen during the interview (2 cases in Bombali, 1 case in Tonkolili and 8 cases in Port Loko)

### Factors associated with incomplete immunisation status

Table 5 shows univariate and multivariable analyses with incomplete immunisation status according to the WHO definition as dependent variable. Following multiple logistic regression, three factors were found to be independently associated with incomplete immunisation status. Compared with the district of Bombali, children residing in Port Loko were more likely to have missed at least one basic vaccination (aOR = 3.47, 95% CI = 2.00-6.06; *p*-value < 0.001). Children whose interviewed caretaker was male were also more likely to be incompletely immunised (aOR = 1.93, 95% CI = 1.03–3.59, *p*-value = 0.039). Compared to children whose caretaker was Christian, those with a Muslim caretaker were more likely to have missed one or more basic vaccinations as well (aOR = 1.94, 95% CI = 1.25–3.02; *p*-value = 0.015). Partly due to categories with few observations, the other factors included in the multivariable analysis showed imprecise estimates with wide 95% CIs, prompting the possibility of no association between these factors and incomplete immunisation status.

**Table 5:** Logistic regression models (univariate and multivariable), output: Incomplete vaccination status (WHO-definition)

Variable ^1^		Univariate models		Multivariable model ^2^	
		Crude OR (95% CI)	*p*-value	Adjusted OR (95% CI)	*p*-value
Child’s age in months (*n* = 720)		0.96 (0.92-1.00)	0.047	0.96 (0.92-1.00)	0.064
Child’s sex	Female (*n* = 363)	1	0.997	1	0.588
	Male (*n* = 357)	1.00 (0.74–1.36)		1.10 (0.77–1.59)	
Caretaker’s age in years (*n* = 718)		0.99 (0.98-1.00)	0.112	1.00 (0.98–1.01)	0.417
Caretaker’s sex	Female (*n* = 624)	1	0.963	**1**	**0.039**
	Male (*n* = 93)	1.01 (0.61–1.70)		**1.93 (1.03–3.59)**	
Caretaker’s highest level of education	Never attended school (*n* = 395)	1	0.575	1	0.928
	Primary (*n* = 78)	0.84 (0.46–1.52)		0.89 (0.47–1.68)	
	Secondary or higher (*n* = 245)	0.83 (0.58–1.19)		0.93 (0.51–1.67)	
Is the caretaker able to read and write?	Illiterate (*n* = 519)	1	0.333	1	0.292
	Partially literate (*n* = 105)	0.78 (0.47–1.28)		0.81 (0.40–1.61)	
	Fully literate (*n* = 96)	0.69 (0.41–1.17)		0.54 (0.25–1.19)	
Caretaker’s main type of income	No salary (n = 79)	1	0.097	1	0.131
	Paid employment (n = 27)	0.28 (0.09–0.89)		0.32 (0.10–0.98)	
	Self-employment (n = 614)	0.86 (0.52–1.40)		0.79 (0.45–1.41)	
Caretaker’s marital status	Married or in union (*n* = 578)	1	0.108	1	0.280
	Single (never married) (*n* = 82)	0.93 (0.52–1.65)		0.82 (0.42–1.58)	
	Separated or divorced (*n* = 24)	0.47 (0.16–1.37)		0.58 (0.17–1.96)	
	Widowed (*n* = 31)	0.34 (0.12–0.96)		0.32 (0.10–1.06)	
Caretaker’s religion	Christian (*n* = 210)	1	< 0.001	**1**	**0.015**
	Muslim (*n* = 508)	2.77 (1.83–4.19)		**1.94 (1.25–3.02)**	
	None (*n* = 2)	4.12 (0.25–68.09)		**1.91 (0.21–17.05)**	
Household head’s sex	Female (*n* = 392)	1	0.755	1	0.9193
	Male (*n* = 327)	1.05 (0.75–1.48)		0.98 (0.67–1.45)	
District	Bombali (*n* = 288)	1	< 0.001	**1**	**< 0.001**
	Tonkolili (*n* = 168)	1.61 (0.87–2.99)		**1.27 (0.63–2.56)**	
	Port Loko (*n* = 264)	4.04 (2.43–6.70)		**3.47 (2.00-6.06)**	
Locality	Rural (*n* = 540)	1	0.086	1	0.677
	Urban (*n* = 180)	1.54 (0.94–2.53)		1.12 (0.65–1.91)	

CI, confidence interval; OR, odds ratio

^1^ The first listed category of each variable was taken as reference value

^2^ Following multiple logistic regression, variables significantly associated with incomplete immunisation status are presented in bold

Appendix 2 includes the results of the logistic regression analyses with incomplete immunisation status following Sierra Leone’s EPI schedule as dependent variable.

### Subgroup analysis: children aged 12–23 months

Results of the subgroup analysis for the 625 enrolled children aged 12–23 months are presented in Appendix 3. Adjusted findings on coverage of individual doses and immunisation status are similar to the previously presented results.

## Discussion

This community-based study was the first survey to assess routine immunisation coverage during the COVID-19 pandemic in Sierra Leone. A low percentage of zero-dose children was reported, yet approximately one in three children had missed at least one basic vaccination and almost half had missed at least one age-appropriate vaccination following the country’s EPI schedule. These findings indicate that too many children are only partially vaccinated and, thereby, exposed to VPDs, disability and premature death.

Coverage of the third DTP (DTP3) dose is often used as an indicator for overall routine immunisation system performance [26]. In our 2021 survey, 86.3% of children had received DTP3. This is lower than the WHO-reported national estimate of 92% that year, but higher than the global DTP3 coverage of 81% and estimates in the neighbouring countries of Guinea (47%) and Liberia (66%) [11, 27]. However, the proportion of vaccinated children in this survey still falls short of the 90% target [28]. Survey findings show that individual vaccine coverage rates were highest for those given at birth and decreased with consecutive EPI appointments at later ages. Accordingly, in the case of multi-dose vaccines including DTP, coverage is highest for the first dose and recedes in subsequent doses. A similar pattern was described by the Sierra Leone DHS in 2019 [9].

The lowest reported coverage was MCV2, administered at the final EPI contact at 15 months. MCV2 coverage is used to evaluate the EPI’s ability to deliver vaccines beyond the first year of life [26]. This survey estimated MCV2 coverage at 40.7% among children aged 16–23 months, which is far below the global coverage of 71% at the end of 2021 [11]. It is also far from the protective threshold of 95% needed to achieve herd immunity and prevent measles outbreaks [29]. Over half of eligible surveyed children had not received the two recommended MCV doses, thus, vaccination catch-up campaigns are warranted. Through the addition of PMC and the recently recommended RTS,S/AS01 malaria vaccine to this final visit at 15 months, renewed emphasis is placed on the need to improve EPI attendance in the second year of life [15, 30]. Strengthening immunisation beyond the first year of life requires implementation of other known strategies as well, including interventions related to community engagement and capacity-building for health workers [31, 32].

Reporting recent vaccination coverage rates is especially relevant in light of the COVID-19 pandemic, which destabilised health systems worldwide [11]. The indirect effects of the pandemic on health service delivery and child health are substantial due to, among others, vaccination campaign interruptions, health resources diversion, supply-chain disruptions, mobility restriction and fear of contagion at health facilities [33–35]. When comparing the coverage rates among a subset of surveyed children aged 12–23 months with the Sierra Leone 2019 DHS report, we found that full immunisation coverage improved in all three districts and that there was no increase in the proportion of zero-dose children [9]. However, it remains unclear whether such differences are due to actual differences in population coverage, or due to differences in survey design and implementation.

This study also found that full immunisation coverage was lower Port Loko than in Bombali and Tonkolili, and around 5% of children in this district were zero-dose. Similarly, Port Loko had the lowest immunisation coverage at district level according to the 2019 DHS in Sierra Leone [9]. Together, these findings suggest possible structural difficulties in this district in reaching all children with immunisation efforts. Underlying challenges on both the demand- and the supply-side should be investigated and addressed in Port Loko.

Children whose interviewed caretaker was male were more likely to have missed at least one routine vaccination. Previous studies conducted in Sub-Saharan Africa identified low male involvement in and poor knowledge of routine childhood immunisation activities as barriers to vaccination uptake [23, 36, 37]. Educating and involving men in matters related to their child’s health can help improve childhood immunisation coverage [38].

In this study, we observed that children with Muslim caretakers were less likely to have received all routine vaccinations. This is in agreement with a previous report showing a lower full immunisation coverage among Muslims than Christians in Sierra Leone [39]. Religion is a complex, multidimensional construct that has long been considered a social determinant of health [40–42]. Understanding religious influences on health-seeking behaviour is crucial to explore possible roots of health inequities [43]. In Sierra Leone, where 77% of the population is Muslim and 22% is Christian, religious leaders have long been involved in mobilising communities and raising awareness on childhood immunisation [44, 45]. This approach has been supported by findings from a previous study in Sierra Leone that observed a higher immunisation confidence and uptake among those who received related information from faith leaders [46]. The findings of the present survey highlight the continued call for involving religious and other influential community leaders in health interventions to promote immunisation uptake. Moreover, tailored interventions may be informed by additional socio-anthropological research that explores religion as a determinant of vaccination uptake in the surveyed area.

It is important to note that although some well-documented determinants of vaccination uptake, such as education and income levels of the caretaker, were not significantly associated with incomplete immunisation status in this survey, they should continue to shape context-specific intervention strategies that strive to close immunisation gaps [22, 23].

Due to frequent incompleteness and inaccuracy of official estimates, vaccination coverage surveys are considered a reliable complement to routine health facility data [47–49]. In this study, we tried to minimise known challenges of coverage surveys [50]. A multi-stage cluster sampling approach was used to select a representative sample. Field workers were trained beforehand and supervised during data collection to maximise data consistency. Furthermore, the use of electronic devices and strict data quality checks during data collection improved the validity of our findings. Lastly, the 1.1% non-response rate among eligible children was much lower than the estimated 10% used to calculate the survey’s sample size, minimising the potential for non-response bias at this level.

A possible study limitation is the fact that some potentially relevant determinants of immunisation uptake were not collected in the survey, such as a wealth and assets index, place of child birth and number of children in the household [22, 23, 51, 52]. Secondly, our estimate of MCV2 coverage does not conform to the target population aged 24–35 months as recommended by the WHO reference manual on vaccination coverage surveys [24]. Instead, our estimate amongst children aged 16–23 months considered a possible delay of one month. Moreover, even though previous studies support the accuracy of caretaker recollection and the majority of caretakers had their child’s vaccination card at hand, a potential recall bias may be present [53–56]. Considering a total of eight EPI visits, our estimates could have been biased by the survey’s reliance on caretakers’ memory in one out of every five interviews. This study has therefore presented individual vaccination coverage rates according to source of information. Lastly, clusters were selected using the 2015 census. Two selected clusters were outside project areas and therefore replaced by backup clusters, leading to potential underrepresentation of part of the area, and thus to potential non-response bias.

## Conclusion

Around one in three surveyed children in selected districts of Sierra Leone had missed at least one basic vaccination and more than half of eligible children had not received the recommended two doses of a measles-containing vaccine. Although full immunisation coverage at district level did not drop below pre-pandemic coverage, too many under-vaccinated children remain at risk of preventable morbidity and mortality. These findings highlight the need for supplementary immunisation activities to ensure improved and equitable vaccination uptake. Identified geographic and cultural barriers to immunisation should be investigated and addressed through tailored strategies to reach under-immunised children. Potential approaches to reduce coverage gaps include additional immunisation efforts in Port Loko, promoting male involvement in matters related to child health, and involving religious leaders to advocate for equal access to life-saving vaccines for all.

### Electronic supplementary material

Below is the link to the electronic supplementary material.

Appendix 1: Survey weight methodology

Appendix 2: Logistic regression models (univariate and multivariable), output: Incomplete vaccination status (following Sierra Leone’s EPI schedule)

Appendix 3: Subgroup analysis: children aged 12 - 23 months

## Data Availability

The datasets generated during the current household survey and analysed in this study are available from the corresponding author on reasonable request.

## Abbreviations

COVID-19: Coronavirus disease 2019
BCG: Bacille Calmette-Guérin
DHS: Demographic and Health Survey
DTP: Diphtheria-pertussis-tetanus
EPI: Expanded Programme on Immunisation
HepB: Hepatitis B
HHS: Household survey
Hib: Haemophilus influenzae type b
IPTi: Intermittent Preventive Treatment in infants
IPV: Inactivated polio vaccine
MCV: Measles-containing vaccine
MULTIPLY: MULTIple doses of IPTi Proposal: a Lifesaving high Yield intervention
OPV: Oral polio vaccine
PMC: Perennial malaria chemoprevention
U2: Under two years of age
UNICEF: United Nations Children’s Fund
VPD: Vaccine-preventable disease
WHO: World Health Organization
