# Supplementary material for: Immunisation coverage and factors associated with incomplete immunisation in children under two during the COVID-19 pandemic in Sierra Leone
Source: BMC Public Health. 2024 Jan 10;24:143. doi: 10.1186/s12889-023-17534-2 (PMC10777622; doi:10.1186/s12889-023-17534-2)
Supplement: Supplementary file 1 — Appendix 1: Survey weight methodology [file 12889_2023_17534_MOESM1_ESM.docx]

Appendix 1. Survey weight methodology

Prior to statistical analysis, survey weights were applied to all records to account for the survey’s multi-stage cluster sampling design. Using data collected by Statistics Sierra Leone during the 2015 national census, weights for the three stages of sample selection were calculated as follows:^1^

$$weight\_1=\frac{1}{p1} weight\_2=\frac{1}{p2} weight\_3=\frac{1}{p3}$$

Where p1 is the probability of being included in the sample for of each of the 60 selected clusters, assuming a systematic random sampling with probability proportional to cluster size:

$$p1= \frac{total population in a specific cluster*60}{total population in the survey area}$$

p2 is the probability of being included in the sample for each household, knowing the total number of households in each cluster and assuming a random selection of 12 households among them:

$$p2=\frac{12}{total number of households in the cluster}$$

and p3 is the probability of a child being randomly selected, among all eligible children in a household. In order to calculate p3, the proportion of the U2 population in Sierra Leone in 2015 (6.26%) was estimated using data on the national annual population by age, provided by the United Nations World Population Prospects.^2^ Data from 2015 were used to match the year the national census in Sierra Leone was conducted, since the sampling frame for the baseline HHS was sourced from this census.

This estimated proportion of the U2 population was then used to calculate the proportion of children U2 in each cluster in our sampling frame, as well as the average number of children U2 per household. p3 was therefore calculated as:

$$p3=\frac{1}{\frac{total population in a specific cluster*0.0626}{total number of households in the cluster}}$$

Considering that the third sampling stage was only performed when one child had to be randomly selected among all eligible children residing in a selected household, weight_3 was only applied to records belonging to households with more than one eligible child.

Calculated survey weights are available upon reasonable request. Weights were applied to all records using the following Stata syntax:

svyset cluster, fpc(fpc1) weight(weight1) || household, fpc(fpc2) weight(weight2) || child, fpc(fpc3) weight(weight3) singleunit(certainty)

Where fpc stands for finite population correction: fpc1 = 216 (total number of clusters in MULTIPLY project areas); fpc2 = number of households in the cluster; fpc3 = number of eligible children in the household, and where singleunit(certainty) causes strata with one sampling unit to be treated as certainty units.

References:

1. Statistics Sierra Leone. Sierra Leone 2015 Population and Housing Census. Available at: https://www.statistics.sl/index.php/census/census-2015.html
2. United Nations World Population Prospects | Annual population by single age - Both Sexes. Available at: <https://population.un.org/wpp/Download/Standard/Interpolated/>
