# Supplementary material for: Immunisation coverage and factors associated with incomplete immunisation in children under two during the COVID-19 pandemic in Sierra Leone
Source: BMC Public Health. 2024 Jan 10;24:143. doi: 10.1186/s12889-023-17534-2 (PMC10777622; doi:10.1186/s12889-023-17534-2)
Supplement: Supplementary file 2 — Appendix 2: Logistic regression models (univariate and multivariable), output: Incomplete vaccination status (following Sierra Leone’s EPI schedule) [file 12889_2023_17534_MOESM2_ESM.docx]

Appendix 2. Logistic regression models (univariate and multivariable), output: Incomplete vaccination status (following Sierra Leone’s EPI schedule)

| **Variable ^1^** | | | **Univariate models** | | **Multivariable model ^2^** | |
| --- | --- | --- | --- | --- | --- | --- |
|  |  |  | **Crude OR (95% CI)** | **p-value** | **Adjusted OR (95% CI)** | **p-value** |
| Child’s age in months (*n*=720) | | | 0.96 (0.92-1.00) | 0.041 | **0.95 (0.91-1.00)** | **0.035** |
| Child’s sex | Female (*n*=363) | | 1 | 0.360 | 1 | 0.910 |
|  | Male (*n*=357) | | 0.88 (0.67-1.16) |  | 0.98 (0.71-1.35) |  |
| Caretaker’s age in years (*n*=718) | |  | 1.00 (0.99-1.01) | 0.552 | 1.01 (0.99-1.02) | 0.450 |
| Caretaker’s sex | Female (*n*=624) | | 1 | 0.571 | 1 | 0.068 |
|  | Male (*n*=93) | | 1.14 (0.72-1.78) |  | 1.66 (0.96-2.86) |  |
| Caretaker’s highest level of education | Never attended school (*n*=395) | | 1 | 0.022 | 1 | 0.343 |
|  | Primary (*n*=78) | | 1.17 (0.71-1.93) |  | 1.37 (0.77-2.42) |  |
|  | Secondary or higher (*n*=245) | | 0.66 (0.47-0.92) |  | 0.84 (0.46-1.52) |  |
| Is the caretaker able to read and write? | Illiterate (*n*=519) | | 1 | 0.018 | 1 | 0.210 |
|  | Partially literate (*n*=105) | | 0.61 (0.39-0.94) |  | 0.66 (0.35-1.22) |  |
|  | Fully literate (*n*=96) | | 0.54 (0.33-0.88) |  | 0.50 (0.23-1.09) |  |
| Caretaker’s main type of income | No salary (*n*=79) | | 1 | 0.042 | 1 | 0.246 |
|  | Paid employment (*n*=27) | | 0.54 (0.20-1.46) |  | 0.64 (0.23-1.82) |  |
|  | Self-employment (*n*=614) | | 1.38 (0.85-2.24) |  | 1.26 (0.74-2.14) |  |
| Caretaker’s marital status | Single (never married) (*n*=82) | | 1 | 0.010 | **1** | **0.013** |
|  | Married or in union (*n*=578) | | 1.25 (0.76-2.07) |  | **0.71 (0.40-1.26)** |  |
|  | Separated or divorced (*n*=24) | | 0.55 (0.19-1.59) |  | **0.44 (0.14-1.35)** |  |
|  | Widowed (*n*=31) | | 0.26 (0.09-0.74) |  | **0.14 (0.04-0.45)** |  |
| Caretaker’s religion | Christian (*n*=210) | | 1 | <0.001 | 1 | 0.105 |
|  | Muslim (*n*=508) | | 2.11 (1.47-3.03) |  | 1.52 (1.03-2.26) |  |
|  | None (*n*=2) | | 2.04 (0.13-33.25) |  | 0.77 (0.08-7.50) |  |
| Household head’s sex | Female (*n*=392) | | 1 | 0.070 | 1 | 0.315 |
|  | Male (*n*=327) | | 1.34 (0.98-1.83) |  | 1.21 (0.83-1.75) |  |
| District | Bombali (*n*=288) | | 1 | <0.001 | **1** | **0.002** |
|  | Tonkolili (*n*=168) | | 1.89 (1.08-3.31) |  | **1.69 (0.93-3.07)** |  |
|  | Port Loko (*n*=264) | | 2.76 (1.76-4.31) |  | **2.75 (1.62-4.68)** |  |
| Locality | Rural (*n*=540) | | 1 | 0.370 | 1 | 0.961 |
|  | Urban (*n*=180) | | 1.23 (0.78-1.95) |  | 0.99 (0.57-1.72) |  |

CI, confidence interval; OR, odds ratio.

^1^ The first listed category of each variable will be taken as reference value.

^2^ Following multiple logistic regression, variables significantly associated with incomplete immunisation status are presented in bold.
