# Supplementary material for: Immunisation coverage and factors associated with incomplete immunisation in children under two during the COVID-19 pandemic in Sierra Leone
Source: BMC Public Health. 2024 Jan 10;24:143. doi: 10.1186/s12889-023-17534-2 (PMC10777622; doi:10.1186/s12889-023-17534-2)
Supplement: Supplementary file 3 — Appendix 3: Subgroup analysis: children aged 12 - 23 months [file 12889_2023_17534_MOESM3_ESM.docx]

Appendix 3. Subgroup analysis: children aged 12 – 23 months

This subgroup analysis includes children aged 12 – 23 months and was performed to conform to the eligibility criteria recommended by the WHO reference manual on vaccination coverage cluster surveys. This manual recommends to include survey participants aged 12 – 23 months if the final primary vaccination is at 9 months of age, allowing for a delay of three months.

Vaccination coverage rates among this subgroup are comparable to those among the complete sample, as presented in this paper (Table 3.1 and 3.2). Following multiple logistic regression, both religion and district were still significantly associated with incomplete immunisation status according to the WHO definition (Table 3.3). However, the sex of the interviewed caretaker was no longer significant in the multivariable model for this subgroup.

Table 3.1. Immunisation status of children aged 12 – 23 months in project districts in Sierra Leone

|  | **Survey districts** | | |  |
| --- | --- | --- | --- | --- |
|  | **Bombali**  n/N (%); [95% CI] | **Tonkolili**  n/N (%); [95% CI] | **Port Loko**  n/N (%); [95% CI] | **Survey area**  n/N (%); [95% CI] |
| **Fully immunised** (according to WHO definition) | 204/255 (80.0);  [72.2-86.1] | 103/143 (72.0);  [61.0-80.9] | 114/227 (50.2);  [42.4-58.1] | 421/625 (67.4);  [61.6 – 72.6] |
| **Fully immunised** (all age-appropriate vaccines from birth to 9 months following Sierra Leone’s EPI schedule) | 173/255 (67.8);  [59.8-75.0] | 76/143 (53.1);  [41.8-64.2] | 100/227 (44.1);  [36.3-52.1] | 349/625 (55.8);  [50.3-61.2] |
| **Partially immunised** (according to WHO definition) | 50/255 (19.6);  [13.5-27.5] | 40/143 (28.0);  [19.1-39.0] | 104/227 (45.8);  [38.3-53.5] | 194/625 (31.0);  [26.0-36.5] |
| **Partially immunised** (some but not all age-appropriate vaccines from birth to 9 months following Sierra Leone’s EPI schedule) | 81/255 (31.8); [24.6-40.0] | 67/143 (46.9);  [35.8-58.2] | 118/227 (52.0);  [44.2-59.6] | 266/625 (42.6);  [37.3-48.0] |
| **Unimmunised** (zero-dose) | 1/255 (0.4);  [0.1-2.8] | 0/143 (0.0) | 9/227 (4.0); [1.8-8.3] | 10/625 (1.6);  [0.7-3.4] |

Table 3.2. Coverage per EPI contact by source of information

|  |  | **Children aged 12–23 months vaccinated at any time before the HHS, according to:** | | |
| --- | --- | --- | --- | --- |
| **Recommended timing of EPI contact** | **Vaccine** | **Vaccination card  n/N (%)** | **Caretaker’s report  n/N (%)** | **Total**  **n/N (%); [95% CI]** |
| **At birth** | BCG | 492/625 (78.3) | 118/625 (19.0) | 610/625 (97.6); [95.8-98.6] |
|  | OPV 0 | 482/625 (74.0) | 118/625 (19.0) | 600/625 (96.0); [93.4-97.6] |
| **At 6 weeks** | OPV 1 | 475/625 (76.8) | 111/625 (17.6) | 586/625 (93.8); [90.2-96.1] |
|  | DTP-HepB-Hib (Pentavalent) 1 | 478/625 (76.3) | 112/625 (18.1) | 590/625 (94.4); [90.8-96.6] |
|  | Pneumococcal 1 | 477/625 (75.7) | 111/625 (17.8) | 588/625 (94.1); [90.6-96.3] |
|  | Rotavirus 1 | 464/625 (73.5) | 112/625 (17.9) | 576/625 (92.2); [88.4-94.8] |
| **At 10 weeks** | OPV 2 | 463/625 (73.2) | 108/625 (17.5) | 571/625 (91.4); [87.4-94.2] |
|  | DTP-HepB-Hib (Pentavalent) 2 | 467/625 (73.8) | 109/625 (17.5) | 576/625 (92.2); [88.3-94.8] |
|  | Pneumococcal 2 | 458/625 (72.5) | 109/625 (17.5) | 567/625 (90.7); [86.9-93.5] |
|  | Rotavirus 2 | 441/625 (69.2) | 110/625 (17.6) | 551/625 (88.2); [84.0-91.3] |
|  | PMC 1 | 373/625 (58.5) | 128/625 (21.3) | 501/625 (80.2); [74.8-84.6] |
| **At 14 weeks** | OPV 3 | 444/625 (69.9) | 105/625 (16.9) | 549/625 (87.8); [83.7-91.1] |
|  | DTP-HepB-Hib (Pentavalent) 3 | 441/625 (69.3) | 106/625 (16.9) | 547/625 (87.5); [83.5-90.6] |
|  | Pneumococcal 3 | 439/625 (69.2) | 103/625 (16.7) | 542/625 (86.7); [82.7-89.9] |
|  | IPV | 384/625 (60.0) | 112/625 (18.1) | 496/625 (79.4); [74.8-83.3] |
|  | PMC 2 | 403/625 (62.8) | 104/625 (17.4) | 507/625 (81.1); [76.5-85.0] |
| **At 6 months** | Vitamin A | 411/625 (64.7) | 104/625 (16.5) | 515/625 (82.4); [77.5-86.4] |
| **At 9 months** | Yellow fever | 363/625 (56.5) | 85/625 (13.3) | 448/625 (71.7); [66.2-76.6] |
|  | MCV 1 | 357/625 (55.6) | 88/625 (14.0) | 445/625 (71.2); [65.7-76.2] |
|  | PMC 3 | 294/625 (45.6) | 82/625 (12.8) | 376/625 (60.2); [54.7-65.3] |
| **At 12 months** | De-worming^1^ | 259/571 (45.4) | 72/571 (12.6) | 331/571 (58.0); [52.1-63.6] |
|  | Vitamin A^1^ | 260/571 (45.5) | 74/571 (13.0) | 334/571 (58.5) [52.7-64.0] |
| **At 15 months** | MCV 2^2^ | 128/442 (29.0) | 52/442 (11.8) | 180/442 (40.7) [34.5-47.2] |

^1^ Among participants aged 13 months or older, allowing one month of delay.

^2^ Among participants aged 16 months or older, allowing one month of delay.

Table 3.3. Logistic regression models (univariate and multivariable), output: Incomplete vaccination status (WHO-definition)

| **Variable ^1^** | | | **Univariate models** | | **Multivariable model ^2^** | |
| --- | --- | --- | --- | --- | --- | --- |
|  |  |  | **Crude OR (95% CI)** | **p-value** | **Adjusted OR (95% CI)** | **p-value** |
| Child’s age in months (*n*=625) | | | 0.98 (0.93-1.02) | 0.334 | 0.97 (0.93-1.02) | 0.304 |
| Child’s sex | Female (*n*=321) | | 1 | 0.623 | 1 | 0.432 |
|  | Male (*n*=304) | | 1.08 (0.78-1.50) |  | 1.17 (0.79-1.73) |  |
| Caretaker’s age in years (*n*=623) | |  | 0.99 (0.98-1.01) | 0.454 | 1.00 (0.98-1.02) | 0.973 |
| Caretaker’s sex | Female (*n*=541) | | 1 | 0.915 | 1 | 0.093 |
|  | Male (*n*=81) | | 0.97 (0.57-1.64) |  | 1.73 (0.91-3.27) |  |
| Caretaker’s highest level of education | Never attended school (*n*=351) | | 1 | 0.4637 | 1 | 0.981 |
|  | Primary (*n*=71) | | 0.84 (0.46-1.54) |  | 0.98 (0.50-1.91) |  |
|  | Secondary or higher (*n*=202) | | 0.79 (0.55-1.15) |  | 0.94 (0.51-1.75) |  |
| Is the caretaker able to read and write? | Illiterate (*n*=460) | | 1 | 0.287 | 1 | 0.238 |
|  | Partially literate (*n*=90) | | 0.77 (0.46-1.28) |  | 0.76 (0.37-1.58) |  |
|  | Fully literate (*n*=75) | | 0.64 (0.36-1.16) |  | 0.48 (0.20-1.14) |  |
| Caretaker’s main type of income | No salary (*n* =69) | | 1 | 0.220 | 1 | 0.270 |
|  | Self-employment (*n* =535) | | 0.80 (0.47-1.36) |  | 0.71 (0.38-1.32) |  |
|  | Paid employment (*n* =19) | | 0.31 (0.08-1.17) |  | 0.34 (0.09-1.30) |  |
| Caretaker’s marital status | Single (never married) (*n*=62) | | 1 | 0.110 | 1 | 0.085 |
|  | Married or in union (*n*=509) | | 1.01 (0.54-1.90) |  | 0.67 (0.33-1.34) |  |
|  | Separated or divorced (*n*=22) | | 0.43 (0.12-1.57) |  | 0.40 (0.10-1.54) |  |
|  | Widowed (*n*=27) | | 0.34 (0.10-1.11) |  | 0.22 (0.06-0.73) |  |
| Caretaker’s religion | Christian (*n*=186) | | 1 | <0.001 | **1** | **0.011** |
|  | Muslim (*n*=437) | | 2.82 (1.80-4.41) |  | **2.11 (1.31-3.40)** |  |
|  | None (*n*=2) | | 4.47 (0.27-74.48) |  | **1.98 (0.22-17.58)** |  |
| Household head’s sex | Female (*n*=345) | | 1 | 0.781 | 1 | 0.838 |
|  | Male (*n*=279) | | 1.05 (0.72-1.54) |  | 1.05 (0.67-1.64) |  |
| District | Bombali (*n*=255) | | 1 | <0.001 | **1** | **<0.001** |
|  | Tonkolili (*n*=143) | | 1.55 (0.80-3.01) |  | **1.22 (0.56-2.65)** |  |
|  | Port Loko (*n*=227) | | 3.96 (2.32-6.79) |  | **3.54 (1.98-6.32)** |  |
| Locality | Rural (*n*=470) | | 1 | 0.138 | 1 | 0.726 |
|  | Urban (*n*=155) | | 1.48 (0.88-2.51) |  | 1.11 (0.60-2.06) |  |

CI, confidence interval; OR, odds ratio.

^1^ The first listed category of each variable will be taken as reference value.

^2^ Following multiple logistic regression, variables significantly associated with incomplete immunisation status are presented in bold.

Table 3.4. Logistic regression models (univariate and multivariable), output: Incomplete vaccination status (following Sierra Leone’s EPI schedule)

| **Variable ^1^** | | | **Univariate models** | | **Multivariable model ^2^** | |
| --- | --- | --- | --- | --- | --- | --- |
|  |  |  | **Crude OR (95% CI)** | **p-value** | **Adjusted OR (95% CI)** | **p-value** |
| Child’s age in months (*n*=625) | | | 0.97 (0.92-1.02) | 0.196 | 0.97 (0.92-1.02) | 0.176 |
| Child’s sex | Female (*n*=321) | | 1 | 0.571 | 1 | 0.957 |
|  | Male (*n*=304) | | 0.92 (0.68-1.24) |  | 0.99 (0.70-1.39) |  |
| Caretaker’s age in years (*n*=623) | |  | 1.00 (0.99-1.01) | 0.810 | 1.01 (0.99-1.03) | 0.186 |
| Caretaker’s sex | Female (*n*=541) | | 1 | 0.599 | 1 | 0.119 |
|  | Male (*n*=81) | | 1.13 (0.71-1.82) |  | 1.58 (0.89-2.82) |  |
| Caretaker’s highest level of education | Never attended school (*n*=351) | | 1 | 0.008 | 1 | 0.136 |
|  | Primary (*n*=71) | | 1.28 (0.77-2.14) |  | 1.60 (0.88-2.91) |  |
|  | Secondary or higher (*n*=202) | | 0.62 (0.43-0.88) |  | 0.80 (0.44-1.47) |  |
| Is the caretaker able to read and write? | Illiterate (*n*=460) | | 1 | 0.020 | 1 | 0.220 |
|  | Partially literate (*n*=90) | | 0.63 (0.40-0.99) |  | 0.71 (0.38-1.34) |  |
|  | Fully literate (*n*=75) | | 0.48 (0.28-0.84) |  | 0.48 (0.21-1.10) |  |
| Caretaker’s main type of income | No salary (*n* =69) | | 1 | 0.153 | 1 | 0.536 |
|  | Self-employment (*n* =535) | | 1.29 (0.78-2.15) |  | 1.13 (0.64-2.01) |  |
|  | Paid employment (*n* =19) | | 0.56 (0.18-1.74) |  | 0.64 (0.18-2.26) |  |
| Caretaker’s marital status | Single (never married) (*n*=62) | | 1 | 0.012 | **1** | **0.011** |
|  | Married or in union (*n*=509) | | 1.31 (0.75-2.30) |  | **0.68 (0.36-1.27)** |  |
|  | Separated or divorced (*n*=22) | | 0.56 (0.18-1.75) |  | **0.37 (0.11-1.30)** |  |
|  | Widowed (*n*=27) | | 0.26 (0.08-0.83) |  | **0.12 (0.03-0.41)** |  |
| Caretaker’s religion | Christian (*n*=186) | | 1 | <0.001 | **1** | **0.047** |
|  | Muslim (*n*=437) | | 2.18 (1.47-3.22) |  | **1.68 (1.12-2.54)** |  |
|  | None (*n*=2) | | 2.21 (0.13-36.15) |  | **0.83 (0.09-8.00)** |  |
| Household head’s sex | Female (*n*=345) | | 1 | 0.113 | 1 | 0.373 |
|  | Male (*n*=279) | | 1.32 (0.93-1.87) |  | 1.20 (0.80-1.82) |  |
| District | Bombali (*n*=255) | | 1 | <0.001 | **1** | **0.003** |
|  | Tonkolili (*n*=143) | | 1.86 (1.05-3.31) |  | **1.60 (0.83-3.08)** |  |
|  | Port Loko (*n*=227) | | 2.68 (1.66-4.32) |  | **2.71 (1.55-4.72)** |  |
| Locality | Rural (*n*=470) | | 1 | 0.420 | 1 | 0.957 |
|  | Urban (*n*=155) | | 1.21 (0.75-1.98) |  | 1.02 (0.55-1.89) |  |

CI, confidence interval; OR, odds ratio.

^1^ The first listed category of each variable will be taken as reference value.

^2^ Following multiple logistic regression, variables significantly associated with incomplete immunisation status are presented in bold.
